# FreeMMIF: interactive multimodal medical image fusion via instruction-aware diffusion

**DOI:** 10.3389/fneur.2026.1802423

**Published:** 2026-06-19

**Authors:** Huizhong Bai, Gang Liu, Bowen Deng, Jiashu Yue, Yu Jiang, Xiaoye Li, Jinyu Li, Xiaohong Mu

**Affiliations:** 1 The First Clinical Medical School, Beijing University of Chinese Medicine, Beijing, China; 2 Department of Mechanical Engineering, Tsinghua University, Beijing, China

**Keywords:** diffusion model, feature re-weighting, image fusion, medical image, vision-language model

## Abstract

As a key technique in clinical diagnosis, multimodal medical image fusion (MMIF) integrates functional and metabolic information to assist diagnosis and enhance disease analysis reliability. However, existing methods typically rely on a single optimization objective, failing to meet clinical demands for flexible, on-the-fly result adjustments. To address this, we propose FreeMMIF, an interactive framework integrating a vision-language model (VLM)-based pseudo-labeling strategy and an instruction-aware diffusion model for task-guided, preference-adaptive fusion. During training, a VLM with specific prompts selects optimal candidates from existing methods as pseudo-ground truths to provide robust supervision without reference images. Subsequently, the diffusion process is modulated via an adaptive feature re-weighting branch, training the model to dynamically coordinate outputs by balancing weighted inputs from source images and pseudo-ground truths. Finally, we employ prompt engineering to construct a weight-generating VLM, allowing physicians to adjust source modality ratios via text instructions for direct control over fusion results. Experimental results demonstrate that FreeMMIF generates diagnostic-quality fusion results that precisely align with clinical intentions. Our code is available at: https://github.com/hzbbucm/FreeMMIF.

## 1 Introduction

Medical imaging plays a pivotal role in clinical settings, enabling the analysis of patient anatomical structures and pathological changes based on specific imaging physics (1, 2). For instance, computed tomography (CT) excels at visualizing dense tissues such as bones and calcifications, while magnetic resonance imaging (MRI) offers superior contrast for soft tissues and organs. However, a single imaging modality is often insufficient to comprehensively characterize a patient's condition, lacking complementary information such as functional metabolism or precise spatial localization. Medical image fusion addresses this limitation by integrating salient features from multiple source images into a single fused representation. This technique has been widely adopted in medical diagnosis and treatment planning; for example, CT/MRI fusion is critical for precise positioning in radiotherapy and neurosurgery, while PET/MRI fusion combines metabolic activity with anatomical context, making it highly suitable for early tumor detection and functional brain imaging.

**Figure 1:**
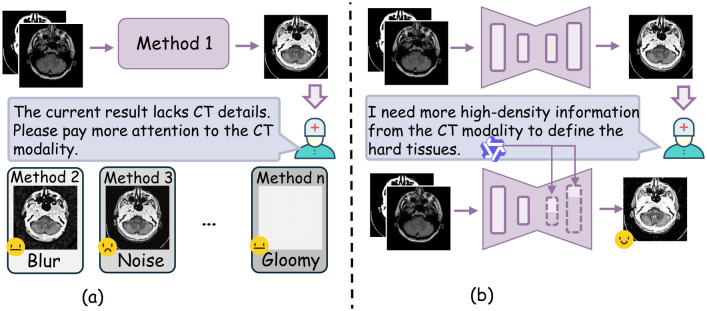
Comparison of the fusion paradigms between **(a)** existing methods and **(b)** FreeMMIF.

Despite remarkable progress, static fusion methods are constrained by fixed optimization objectives, resulting in a “one-size-fits-all” solution space that falls short in complex neurological cases (e.g., glioblastoma or neurotrauma) requiring dynamic transitions between anatomical margin delineation and metabolic assessment. As illustrated in Figure 1, the direct application of existing controllable diffusion models is hindered by the severe scarcity of paired medical text-image datasets. Consequently, a critical research gap remains: the lack of an intuitive interaction mechanism capable of translating abstract clinical intent into real-time, dynamic control over the fusion process. To address this, we propose the instruction-aware FreeMMIF framework, which translates clinical intent into feature modulation parameters, thereby achieving preference-adaptive computer-aided diagnosis.

Furthermore, current methods often require complex parameter tuning, specialized model redesign, or retraining to achieve different fusion effects. Clinical practitioners, such as doctors, typically lack the engineering expertise to manipulate these complex configurations to obtain their desired results. Therefore, there is an urgent need to establish an intuitive interaction mechanism–such as natural language instructions—that bridges the gap between technical model parameters and clinical diagnostic intent, enabling physicians to directly control the fusion process.

In recent years, diffusion models have emerged as a dominant technology in image generation, capable of producing high-fidelity images that align with human perception. However, in the field of medical image fusion, applying diffusion models is challenging due to the scarcity of high-quality text-image paired datasets and established training schemes. Consequently, existing diffusion-based fusion methods struggle to directly align textual instructions with image features. Thus, a key research question remains: how to leverage the powerful generative capabilities of diffusion models to force the network to explore a broader solution space and generate diverse fusion results, thereby increasing their clinical utility.

The FreeU (3) offers a potential solution by controlling the skip connections and backbone feature weights of diffusion models to balance high-frequency and low-frequency information. While effective, FreeU is primarily designed for the inference phase of large-scale pre-trained generative models (e.g., stable diffusion (4)) that inherently possess high diversity. In contrast, existing diffusion-based medical image fusion models are typically trained to be deterministic, making them unsuitable for dynamic adjustment via feature modulation alone. Therefore, adapting feature modulation techniques to dynamically control a specialized medical image fusion diffusion model represents a promising direction to enhance diagnostic flexibility.

To address this, we propose the instruction-aware FreeMMIF framework, which translates clinical intent into feature modulation parameters, thereby achieving preference-adaptive visualization to assist computer-aided diagnosis, rather than acting as a definitive clinical endpoint. FreeMMIF generates fusion results with varying information dominance from a single pair of source images and supports text-controlled dynamic adjustment based on user prompts. Specifically, to obtain diverse and controllable fusion targets from limited modality inputs, we first utilize a vision-language model (VLM) to search for optimal fusion candidates from existing methods that exhibit distinct modality advantages. Then, we mix the source images with these search results and adjust the mixing ratios to create multiple constrained targets. Subsequently, during the training phase, we modulate the feature weight ratios to correspond with these diverse targets, training the model to generate different fusion results based on specific weight configurations. Finally, during deployment, we employ a VLM with preset prompt engineering to interpret user input text and generate corresponding weights, thereby yielding fusion results that precisely meet user requirements.

The main contributions of this work are summarized as follows:

- We propose FreeMMIF, a pioneering interactive multimodal medical image fusion framework. Unlike conventional methods that yield a static solution, FreeMMIF incorporates instruction-aware diffusion to enable language-driven, controllable fusion. This paradigm shift allows for the dynamic adjustment of fusion results, flexibly accommodating diverse clinical diagnostic requirements within a single unified model.
- We design a novel VLM-guided pseudo-labeling strategy to overcome the lack of paired text-image datasets in medical fusion. By leveraging a vision-language model with specific prompts to select optimal candidates from existing methods and synthesizing them with source images, we construct a robust, preference-aligned supervision scheme without relying on ground truth reference images.
- We introduce an adaptive feature re-weighting mechanism based on the principles of FreeU, tailored for medical image fusion. We bridge the gap between clinical text and model parameters by employing a weight-generating VLM that translates textual instructions into modulation coefficients. This allows the diffusion model to dynamically balance the high-and-low frequency information from different modalities (e.g., CT bone structures vs. MRI soft tissues) during the generation process.
- Comprehensive experiments demonstrate that FreeMMIF achieves state-of-the-art performance in both objective metrics and visual quality. More importantly, the framework exhibits precise semantic alignment with physician instructions, providing a highly interpretable and interactive tool that significantly enhances the reliability and flexibility of computer-aided diagnosis.

## 2 Related work

### 2.1 Medical image fusion

Medical image fusion aims to integrate complementary information from different modalities to provide comprehensive visualization, and it has been successfully applied in clinical diagnosis, surgical navigation, and radiotherapy planning (5, 6). (7) noted that the widespread adoption of Picture Archiving and Communication Systems (PACS) enables the storage and display of complementary multimodal images, driving the need for spatial co-registration to increase diagnostic accuracy. They emphasized that voxel-based matching algorithms optimize the similarity between images, facilitating their synergistic clinical use. Furthermore, (8) proposed an integrated framework combining Text-Based (TBIR) and Content-Based Image Retrieval (CBIR) to overcome the limitations of handling heterogeneous medical images. By incorporating a modified ant colony optimization mechanism with relevance feedback. However, while current PACS platforms provide essential image management, retrieval, and basic linear overlay tools, they lack the advanced, non-linear deep feature synthesis required to dynamically and optimally fuse complex multimodal information without manual interference. To address these deeper fusion needs, (9) proposed MATR, an unsupervised multiscale adaptive Transformer fusion method, which significantly enhances global semantic extraction capabilities by combining adaptive convolution with Transformer technology. (10) proposed an unsupervised enhanced medical image fusion network combining surface-level and deep-level constraints, effectively improving the chrominance information and alleviating the mosaic effect in functional images by utilizing high-quality details from structural images. (11) proposed an efficient fusion method based on joint bilateral filter two-layer decomposition, utilizing a novel local gradient energy operator and the l1-max rule to process the structure layer and energy layer, respectively. (12) proposed an unsupervised two-stage medical image fusion framework combining Swin Transformer and CNN, which effectively balances the extraction of global context and local detailed information by designing a residual Swin-Convolution fusion module and an adaptive weight block. (13) effectively overcame data distribution discrepancies by introducing adversarial learning in the feature domain to extract key features from source images, combined with a decoder supervised by natural images through an alternating training strategy. (14) proposed an end-to-end unsupervised adversarial network comprising a generator and dual-symmetric discriminators, which effectively eliminated checkerboard artifacts and enhanced texture detail preservation by adopting an improved U-Net architecture. However, existing methods typically generate fixed fusion results oriented towards a single optimization objective, making them difficult to adapt to practical clinical diagnosis scenarios.

### 2.2 Controllable diffusion model

Controllable diffusion models are capable of controlling image generation to align with human preferences based on prompts provided by humans, such as text, layouts, and sketches. Zheng et al. (15) proposed a highly controllable diffusion model named LayoutDiffusion, which effectively improves multimodal fusion accuracy and spatial information control capabilities by introducing a layout fusion module and an object-aware cross-attention mechanism. Vecchio et al. (16) proposed a unified material generation and editing method based on diffusion models named MatFuse, which achieves fine-grained control by integrating multiple condition inputs such as color, sketches, and text, and utilizes a multi-encoder compression model to learn disentangled latent representations to support map-level material editing. Inoue et al. (17) proposed a unified generation framework named LayoutDM based on discrete state-space diffusion models for controllable layout generation tasks, which progressively infers noiseless layouts through a modality-wise discrete diffusion process and achieves flexible conditional constraints using masking or logit adjustment mechanisms during the inference stage. Zeng et al. (18) proposed a controllable mind visual diffusion model named CMVDM, which accurately extracts key features from fMRI data through attribute alignment and assistant networks, and introduces a control model and residual blocks to guide image synthesis. Wang et al. (19) proposed a novel disentanglement framework combining CLIP and diffusion models, which explicitly extracts content information and implicitly learns style features using CLIP loss, achieving flexible and controllable disentanglement while obtaining generation results superior to state-of-the-art techniques. Si et al. (3) proposed a training-free, plug-and-play method named FreeU, which effectively balances the advantages of skip connections and backbone feature maps by strategically re-weighting their contributions during the inference stage, significantly improving the generation quality of existing diffusion models by simply adjusting scaling factors. However, the field of medical image fusion currently lacks controllable fusion methods, making it difficult to match the immediate diagnostic needs of doctors for observing different modalities.

### 2.3 Vision language model

Vision-language models accept multimodal text-image inputs and perform cross-modal feature alignment and deep semantic reasoning to ultimately produce text outputs (20, 21). Dai et al. (22) proposed the InstructBLIP model incorporating an instruction-aware query transformer, which effectively extracts instruction-relevant features by constructing an instruction tuning collection covering 26 datasets. DeepSeek (23) combines multi-head latent attention (MLA) with a mixture-of-experts (MoE) architecture, and pioneers an auxiliary-loss-free strategy for load balancing and a multi-token prediction objective. Its comprehensive performance surpasses other open-source models and rivals current top-tier closed-source models. Bai et al. (24) introduced the comprehensive large language model series named Qwen, covering models ranging from general foundation models to chat models fine-tuned with reinforcement learning from human feedback (RLHF), as well as specialized models optimized for coding and mathematics. This series not only demonstrates superior downstream task performance and advanced tool-use planning capabilities, but its specialized versions also significantly outperform similar open-source models in code generation and mathematical reasoning, approaching the level of top-tier closed-source models. Achiam et al. (25) presented the large-scale multimodal model GPT-4, which not only supports image and text inputs but also achieves human-level excellence on professional academic benchmarks through post-training alignment. The Google team (26) launched the Gemini series models comprising Ultra, Pro, and Nano sizes, achieving full coverage from complex cloud-based reasoning to memory-constrained on-device scenarios. Xu et al. (27) addressed the lack of systematic reasoning capabilities in existing vision-language models when handling complex visual question answering by proposing an autonomous multi-stage reasoning model named LLaVA-CoT and a corresponding 100k structured dataset. Existing VLMs can effectively understand human instructions, and combining VLMs with image fusion can effectively enhance diagnostic accuracy.

### 2.4 Clinical applications of medical image fusion

Medical image fusion has been widely applied in clinical fields such as radiotherapy planning, surgical navigation, and tumor staging. However, different clinical tasks impose distinct information requirements on fusion results. In radiotherapy planning, CT-MRI fusion has become a key technique for precise delineation of tumor target volumes. Mokhtar et al. (28) found that in radiotherapy planning for Glioblastoma brain tumor, target volume delineation using fused images yielded more comprehensive coverage compared to CT alone, significantly improving the accuracy of target localization. Yang et al. (29) demonstrated that in preoperative planning for skull base communicating tumors, CT-MRI fusion combined with three-dimensional reconstruction enables precise assessment of the spatial relationships between the tumor and craniofacial bones, blood vessels, and brain tissue, thereby reducing the difficulty that surgeons face when mentally integrating images of complex anatomical structures. In a retrospective study, (30) found that the use of CT/MRI images effectively shortened the operative time for patients with lumbar spinal stenosis undergoing percutaneous endoscopic lumbar decompression, and achieved better postoperative recovery outcomes. Nevertheless, even within the same patient, the requirements for fusion results vary considerably across different stages of diagnosis and treatment: during initial diagnosis, oncology patients require imaging that emphasizes structural information, whereas tumor staging relies more heavily on highlighting metabolic information, and treatment response evaluation necessitates differentiation between viable tumor and post-treatment changes (31). Current fusion methods typically produce only a single fixed output and cannot dynamically adjust the display emphasis according to the specific clinical objective, which limits the practical utility of fusion techniques in clinical practice. There is an urgent need for an adaptive fusion approach capable of dynamic adjustment based on specific diagnostic requirements to meet the demands of multi-stage, multi-task clinical image fusion.

## 3 Method

This section provides a detailed description of the FreeMMIF implementation pipeline, including its architecture and training scheme.

### 3.1 Overview

**Figure 2:**
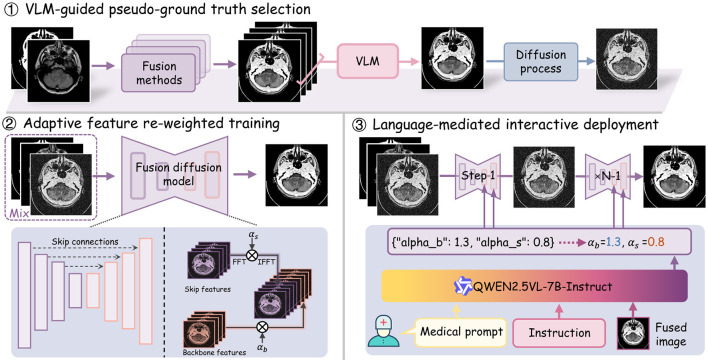
The overall framework of FreeMMIF. The pipeline consists of three stages: (1) VLM-guided pseudo-ground truth selection, which synthesizes robust training targets; (2) Adaptive feature re-weighted training, where the diffusion model learns dynamic feature modulation; and (3) Language-mediated interactive deployment, which translates clinical instructions into parameter weights to dynamically guide the fusion process.

In this paper, we propose FreeMMIF, an interactive multimodal medical image fusion framework capable of generating controllable results aligned with clinical intentions. As illustrated in Figure 2, the proposed framework consists of three distinct phases: VLM-guided pseudo-ground truth selection: (1) A data construction phase where a vision-language model selects optimal fusion candidates from existing methods to construct diverse pseudo-ground truths. (2) Adaptive feature re-weighted diffusion training: During the training phase, a diffusion model is trained using an adaptive feature modulation mechanism to reconstruct multi-objective fusion targets synthesized from pseudo-ground truths and source images. (3) Instruction-driven inference: A deployment phase where a weight-generating VLM translates clinician textual instructions into modulation coefficients to control the generation process.

In a practical neurological clinical workflow, FreeMMIF is integrated into the post-acquisition diagnostic workstation. First, the acquired multimodal images are loaded into the system. Next, depending on the specific diagnostic stage–such as structural assessment for surgical navigation or metabolic evaluation for radiotherapy planning–the physician inputs specific textual instructions. FreeMMIF then translates these text instructions into corresponding feature-reweighting parameters to dynamically generate customized fused images on-the-fly, directly supporting the subsequent clinical decision-making process.

### 3.2 VLM-guided pseudo-ground truth selection

Since medical image fusion lacks paired text-image datasets and standard ground truths, we design a self-supervised strategy to construct a training set with diverse preference priors. Given a pair of source images, PET/CT (*I*_*A*_) and MRI (*I*_*B*_), we first apply *N* representative state-of-the-art fusion methods to generate a candidate set $C=\left\{F_{1},F_{2},...,F_{N}\right\}$. To evaluate the quality of these candidates without reference images, we employ a pre-trained VLM as an evaluator. We design specific prompts $P_{\text{eval}}$ covering key diagnostic metrics (e.g., "contrast," "texture details," "artifact presence"). The VLM selects the optimal candidate *F*_best_ that best satisfies the prompt, as defined in Equation 1:


$$\begin{array}{l} F_{best}=\text{VLM}\left(C,P_{\text{eval}}\right). \end{array}$$ (1)

To further expand the solution space and enable continuous control, we do not strictly use *F*_best_ as the sole target. Instead, we synthesize pseudo-ground truths (*I*_pgt_) by dynamically mixing the source images with the selected candidate using randomized mixing ratios. This creates a spectrum of potential fusion results ranging from source-dominant to algorithm-dominant, as formulated in Equation 2:


$$\begin{array}{l} I_{\text{pgt}}=\mu_{1}\cdot \left(\lambda_{1}\cdot I_{A}+\lambda_{2}\cdot I_{B}\right)+\mu_{2}\cdot F_{\text{best}}, \end{array}$$ (2)

where λ_1_ and λ_2_ are randomly sampled coefficients during data preparation. This strategy ensures the model learns to reconstruct images with varying distributions of modal information.

### 3.3 Adaptive feature re-weighted diffusion training

To enable adaptive dynamic adjustment for the diffusion-based fusion model, we construct the adaptive feature re-weighting. This module dynamically modulates the U-Net features to align with the desired fusion preferences.

#### 3.3.1 Conditional diffusion backbone

The backbone is a conditional U-Net, denoted as ϵ_θ_. To guide the denoising process with source information, we employ a channel-concatenation strategy. Specifically, at each timestep *t*, the noisy state *x*_*t*_ and the two source modalities *I*_*A*_ and *I*_*B*_ are concatenated along the channel dimension to form the input, as expressed in Equation 3:


$$\begin{array}{l} Input_{t}=\text{Concat}\left(x_{t},I_{A},I_{B}\right). \end{array}$$ (3)

This allows the network to directly leverage the spatial structure of both anatomical and metabolic modalities to predict the noise ϵ.

#### 3.3.2 Stochastic modality-coupled strategy

To achieve precise controllability, we introduce the frequency-aware adaptive feature modulation (FA-AFRM). As implemented in our architecture, this module intercepts the decoder's feature streams at specific resolution stages and modulates them through two distinct mechanisms: spatial channel scaling for backbone features and spectral low-frequency scaling for skip connections. Recognizing that different network depths encode different semantic levels, we apply modulation only at the pivotal upsampling stages of the U-Net (specifically where channel dimensions are 192 and 96). This prevents excessive signal distortion while effectively steering the generation process.

For the backbone feature maps $b_{l}\in {\mathbb{R}}^{B\times C\times H\times W}$, we apply a partial channel scaling strategy. Instead of scaling the entire tensor, we only modulate the first half of the channels to adjust the structural information flow while preserving the remaining feature diversity, as formulated in Equation 4:


$$\begin{array}{l} b_{l}\left[:,0:C/2\right]=b_{l}\left[:,0:C/2\right]\cdot \alpha_{b}, \end{array}$$ (4)

where α_*b*_ is the control coefficient derived from our modality-coupled strategy. This operation explicitly strengthens or suppresses the structural context provided by the backbone network.

For the skip connections *s*_*l*_, which bridge the encoder and decoder, we employ a Fourier-based filter to selectively control the information transfer. The goal is to modulate the low-frequency semantic components while preserving high-frequency edge details. The process involves three steps. First, the feature map *s*_*l*_ is converted to the frequency domain using the fast Fourier transform (FFT), as shown in Equation 5:


$$\begin{array}{l} S\left(u,v\right)=\text{FFT}\left(s_{l}\right). \end{array}$$ (5)

We construct a frequency mask M centered at the zero-frequency component. The scaling factor α_*s*_ is applied exclusively to a central region defined by a threshold radius *r* (set to 1 in our implementation), while high-frequency components remain unchanged, as defined in Equation 6:


$$\begin{array}{l} S_{\text{mod}}\left(u,v\right)=\{\begin{array}{ll} S\left(u,v\right)\cdot \alpha_{s}, & \text{if}\left(u,v\right)\in {\text{Region}}_{\text{center}}\\ S\left(u,v\right), & \text{otherwise} \end{array}. \end{array}$$ (6)

This effectively amplifies or suppresses the low-frequency energy of the skip connection based on the target modality ratio, without blurring the high-frequency boundaries.

The modulated spectrum is transformed back to the spatial domain via inverse FFT (IFFT), as given in Equation 7:


$$\begin{array}{l} s_{l}^{\prime }=\text{Real}\left(\text{IFFT}\left(S_{\text{mod}}\right)\right). \end{array}$$ (7)

By coupling these two operations with the training-time modality ratios, FreeMMIF learns to dynamically reconfigure the internal information flow–balancing structural guidance from the backbone and semantic details from the skip connections–strictly following the generated weights.

#### 3.3.3 Dual-Mode training objective

To ensure the model possesses both robust reconstruction capabilities and precise responsiveness to control signals, we implement a Dual-Mode Training Strategy. The training flow dynamically switches between two modes based on a probability threshold *p*_coupled_ (set to 0.5):

**Adaptive learning mode** Activated when the random probability is below *p*_coupled_. In this mode, we enforce the stochastic modality-coupled strategy described in Sec. 3.3.2. We first define the dynamic adaptive target $x_{0}^{\text{adapt}}=I_{\text{pgt}}$ for a given iteration based on the sampled ratios λ_*A*_, λ_*B*_. Simultaneously, we explicitly map the modality ratios to the FA-AFRM control weights **W**(λ) = {α_*s*_, α_*b*_}. Based on the inductive bias that skip connections dominate high-frequency details while backbone features control structural semantics, the coupling relationship is defined as in Equation 8:


$$\begin{array}{l} \alpha_{s}=1+\frac{\lambda_{A}}{2},\;\alpha_{b}=0.5+\frac{\lambda_{B}}{2}. \end{array}$$ (8)

The optimization objective $L_{\text{adapt}}$ then forces the model to predict the noise ϵ added to this dynamic target, strictly conditioned on these coupled weights, as expressed in Equation 9:


$$\begin{array}{l} {ℒ}_{\text{adapt}}=E_{t,\epsilon ,\lambda }\left[{\left\|\epsilon -\epsilon_{\theta }(x_{t}^{\text{adapt}},t,W(\lambda ),\text{Concat}(I_{A},I_{B}))\right\|}_{1}\right], \end{array}$$ (9)

where $x_{t}^{\text{adapt}}$ is the noisy state derived from $x_{0}^{\text{adapt}}$, and ||·||_1_ denotes the *L*_1_ loss. By minimizing this objective, the network learns the bijective mapping between the injected instruction weights and the structural dominance of the specific modality mixture.

**Standard reconstruction mode** To prevent the model from overfitting to the synthetic mixing artifacts and to ensure high-quality baseline fusion, we employ a standard reconstruction path. Here, the target is set directly to the optimal candidate selected by the VLM: $x_{0}^{\text{std}}=F_{\text{best}}$.

Crucially, the FA-AFRM parameters are reset to an identity mapping, i.e., **W**_id_ = {α_*s*_ = 1.0, α_*b*_ = 1.0}, effectively disabling the modulation. The corresponding loss $L_{\text{std}}$ ensures the U-Net functions as a standard diffusion model, as defined in Equation 10:


$$\begin{array}{l} {ℒ}_{\text{std}}=E_{t,\epsilon }\left[{\left\|\epsilon -\epsilon_{\theta }(x_{t}^{\text{std}},t,W_{\text{id}},\text{Concat}(I_{A},I_{B}))\right\|}_{1}\right], \end{array}$$ (10)

The final unified training process alternates between these two objectives. This strategy enables FreeMMIF to simultaneously capture the general distribution of high-quality fusion images and master the specific conditional control mechanism.

### 3.4 Instruction-driven inference

We utilize a VLM (Qwen-VL) as a parameter translator. Through prompt engineering, the VLM is instructed to analyze the user's text and quantify the intent into modality dominance ratios λ_*A*_, λ_*B*_. These ratios are then mapped to the inference-time control weights ${\text{W}}_{\text{pred}}=\left\{\alpha_{s}^{\text{pred}},\alpha_{b}^{pred}\right\}$ using the same coupling logic defined in the training phase, as expressed in Equation 11:


$$\begin{array}{l} {\text{W}}_{\text{pred}}={\text{VLM}}_{\text{infer}}\left(\text{User Instruction}\right)\to \left\{\alpha_{s},\alpha_{b}\right\}. \end{array}$$ (11)

We perform the deterministic DDIM sampling to reconstruct the image from pure noise *x*_*T*_. Unlike standard generation, the FA-AFRM module remains active throughout the reverse diffusion process.At each sampling step *t*, the predicted weights **W**_pred_ are injected into the U-Net. The FA-AFRM spatially scales the backbone features and spectrally filters the skip connections based on **W**_pred_. The refined noise prediction is computed as in Equation 12:


$$\begin{array}{l} \epsilon_{t}=\epsilon_{\theta }\left(x_{t},t,{\text{W}}_{\text{pred}},\text{Concat}\left(I_{A},I_{B}\right)\right). \end{array}$$ (12)

The denoised image at step *t*−1 is then derived using the DDIM update rule, as given in Equation 13:


$$\begin{array}{l} x_{t-1}=\sqrt{{\bar{\alpha }}_{t-1}}\left(\frac{x_{t}-\sqrt{1-{\bar{\alpha }}_{t}}\epsilon_{t}}{\sqrt{{\bar{\alpha }}_{t}}}\right)+\sqrt{1-{\bar{\alpha }}_{t-1}}\epsilon_{t}. \end{array}$$ (13)

By consistently applying the frequency-aware modulation at every timestep, FreeMMIF steers the diffusion trajectory to converge closer to the semantic manifold specified by the physician, yielding a fusion result that is both clinically accurate and preference-aligned.

Instruction for VLM-based Pseudo-GT Selection

- **Task Definition:** You will be given four fused images labeled 1–4. Assess only perceptual image quality.
- **Focus (Modality-Specific):**
  – For **CT-PET**: Prioritize *bone structure retention* (CT) and *metabolic signal transfer* (PET).
  – For **MRI-PET**: Prioritize *soft tissue detail retention* (MRI) and *metabolic signal transfer* (PET).
- **Negative Constraints:** Penalize visible artifacts including halos, ringing, color cast, and over/under-exposure.
- **Formatting Rules:** Provide a strict ranking from best to worst using “>” as separators (e.g., 3>1>4>2). No extra words or punctuation.

## 4 Experiments

### 4.1 Setting up

#### 4.1.1 Dataset

To comprehensively evaluate the performance of the proposed FreeMMIF framework, we conducted experiments using the Harvard Medical Dataset for training and extended the validation to include both the Harvard Medical Dataset and the UTFB dataset collected by Siam et al. (32). During the training phase, we adopted a cropping strategy to augment the dataset and ensure robust feature learning. To guarantee the integrity of our evaluation and strictly rule out any data leakage, the dataset split is performed at the independent scan level prior to any preprocessing. Specificall, we selected 160 pairs of CT/MRI images, which were cropped into 4,000 pairs of patches with a resolution of 128 × 128. Similarly, for the functional imaging task, 245 pairs of PET/MRI images were processed into 6,125 pairs of 128 × 128 patches to constitute the training set. To verify the effectiveness and generalization capability of FreeMMIF, the testing set was constructed using data from two distinct sources. We utilized 24 pairs of CT/MRI images and 24 pairs of PET/MRI images from the Harvard Medical Dataset. Furthermore, to assess the model's performance on real-world clinical acquisitions, we included an additional 33 pairs of CT/MRI images from the UTFB dataset as an external test set.

For the PET-MRI fusion task, we adopt the standard YCbCr color space transformation to preserve functional color fidelity. Specifically, the RGB PET images are converted into the YCbCr space, and the fusion network is trained exclusively to fuse the MRI image with the luminance (Y) channel of the PET image. During inference, the fused luminance channel is directly combined with the original chrominance channels (Cb and Cr) from the source PET image before being converted back to RGB, thereby structurally guaranteeing the preservation of the original metabolic color information without distortion.

#### 4.1.2 Training detail

We implement the proposed FreeMMIF using the PyTorch framework on a single RTX 4090 NVIDIA GPU. To optimize the network parameters, we use the Adam optimizer with an initial learning rate of 1 × 10^−4^. The training process consists of 2 × 10^4^ iterations with a batch size of 16. For the diffusion process, we set the total number of timesteps *T* to 1,000, employing a linear variance schedule where β linearly increases from 1 × 10^−6^ to 1 × 10^−2^. Regarding the network architecture, the conditional denoising U-Net is configured with an initial channel capacity of 48. We adopt channel multipliers of [1, 2, 4, 6] and employ 2 residual blocks at each resolution level, with attention mechanisms applied at the 16 × 16 resolution scale to capture global dependencies. Additionally, for the pseudo-ground truth synthesis described in Equation 2, the mixing coefficients are set to μ_1_ = 0.8 and μ_2_ = 0.2, respectively, to effectively balance the retention of source modality information with the optimal candidate features. Furthermore, we employ a specific prompt template labeled “Instruction for VLM-based Pseudo-GT Selection,” which guides the VLM to select the optimal pseudo-ground truth from candidates generated by four representative fusion methods. Furthermore, to eliminate potential bias and instability arising from the use of varying vision-language models, we explicitly standardize the VLM component across our framework. We strictly utilize the Qwen2.5-VL-7B-Instruct model for all VLM-related tasks. For the inference phase, we utilized 4 DDIM steps to ensure efficient and high-quality generation.

#### 4.1.3 Compare methods

To comprehensively evaluate the effectiveness and superiority of FreeMMIF, we conduct comparative experiments against seven representative state-of-the-art fusion methods. These baselines encompass a diverse range of technical architectures, including traditional multi-scale transform methods, convolutional neural networks, Transformer, and generative adversarial networks. Specifically, the comparison methods include DDFM (33), EMMA (34), IFCNN (35), NSST-PAPCNN (36), RCGAN (37), U2Fusion (38), and GIFNet (39).

### 4.2 Results of CT-MRI medical image fusion

This section evaluates the performance of FreeMMIF in fusing CT and MRI images from both qualitative and quantitative perspectives.

#### 4.2.1 Qualitative comparisons

**Figure 3:**
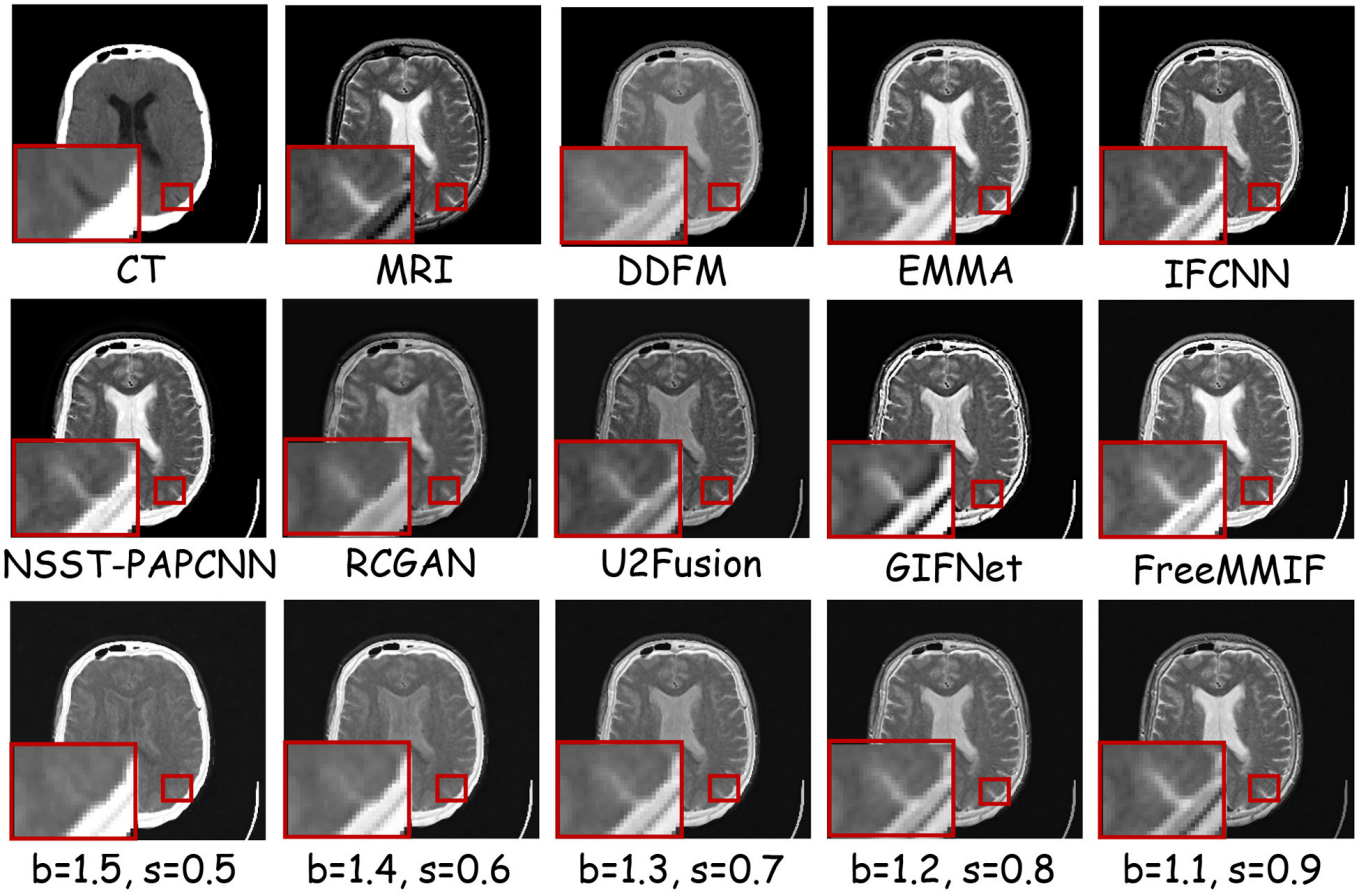
Visual comparison of CT-MRI fusion results on the Harvard Medical Dataset.

**Figure 4:**
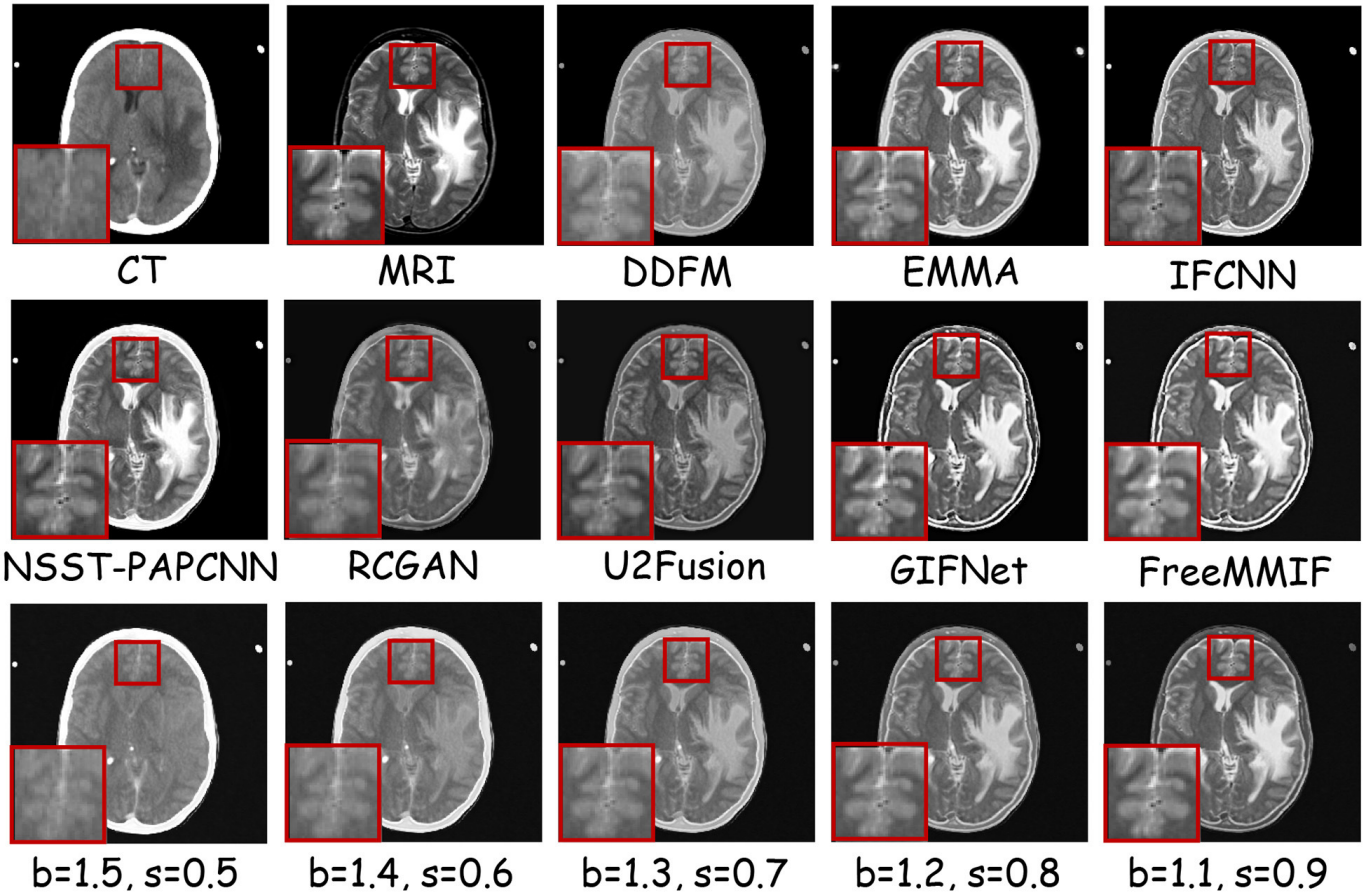
Visual comparison of CT-MRI fusion results.

We compare the fusion results of FreeMMIF with seven state-of-the-art methods on the CT-MRI image fusion task. Figures 3, 4 visually present the fused images obtained by different methods. As observed in the zoomed-in regions, methods such as LRFNet and RCGAN reduce the contrast of high-density structures (e.g., bones), resulting in blurred edges. Although methods like NSST-PAPCNN and EMMA improve the overall brightness, they often introduce artifacts or fail to fully preserve the fine soft tissue textures from MRI images.

In contrast, FreeMMIF effectively extracts and integrates complementary information from the source images. It retains the intact dense bone structures from the CT images while preserving the rich soft tissue details from the MRI images. The fusion results exhibit sharp edges and high contrast, aligning more closely with human visual perception. Simultaneously, FreeMMIF with different parameters provides physicians with more information from the original images, facilitating accurate clinical diagnosis.

**Figure 5:**
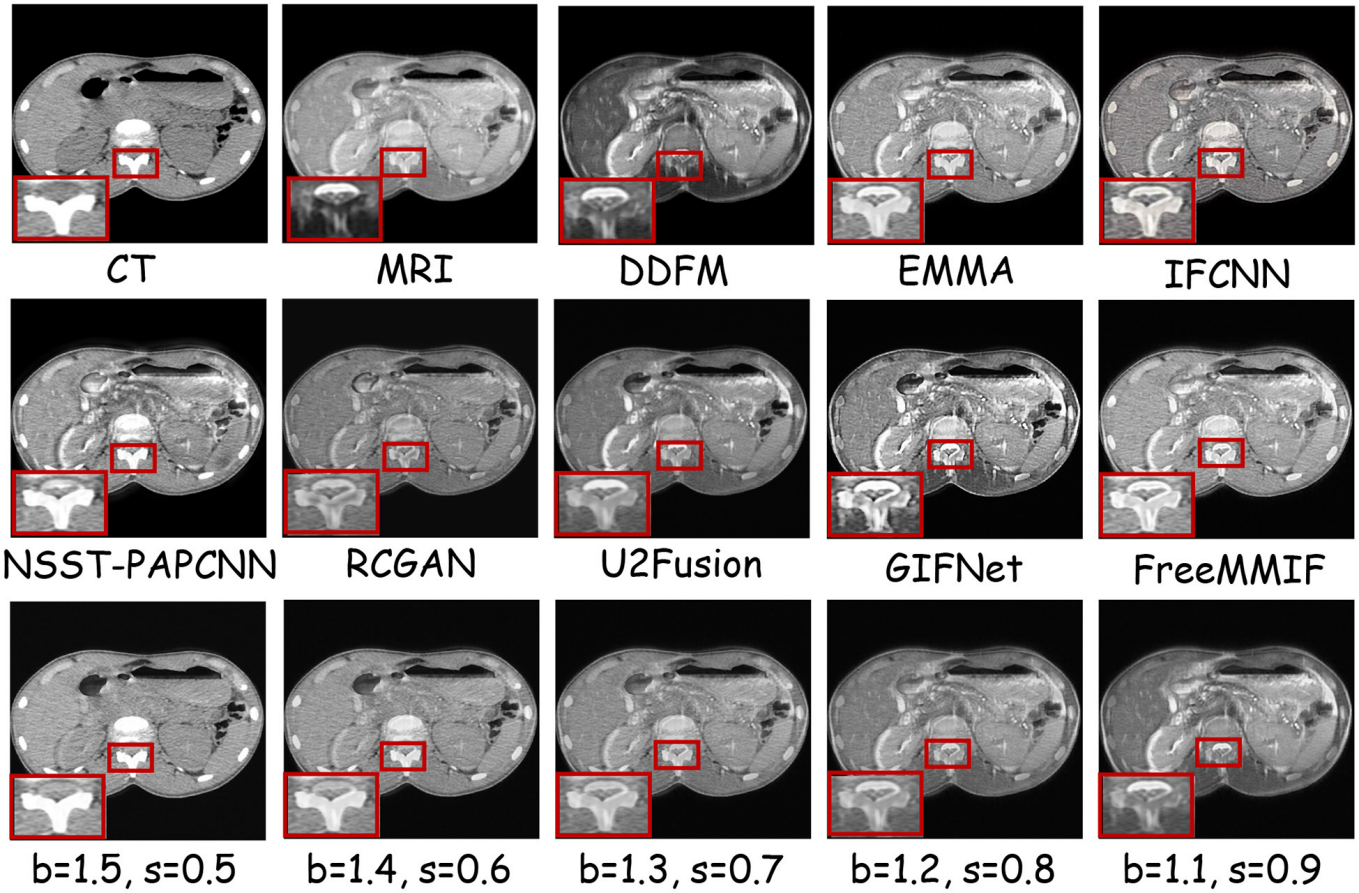
Visual comparison of CT-MRI fusion results.

Furthermore, Figure 5 demonstrates the robustness of our method across different anatomical regions. Whether processing cranial or abdominal structures, FreeMMIF maintains high contrast and rich information content. The results indicate that our proposed instruction-aware mechanism successfully balances complementary information, avoiding the "one-size-fits-all" limitation observed in methods like IFCNN and NSST-PAPCNN. By dynamically adjusting the control weights (e.g., α_*b*_ = 1.1, α_*s*_ = 0.9), FreeMMIF generates images that are not only visually pleasing but also hold significant clinical value for diagnostic tasks.

#### 4.2.2 Quantitative comparisons

We quantitatively evaluate the performance of FreeMMIF using eight standard metrics. Table 1 quantitatively presents the comparison results between FreeMMIF and the other seven fusion methods on the CT-MRI dataset.

**Table 1:** Quantitative comparison of CT-MRI fusion performance on the Harvard Medical Dataset.

	MI	EN	VIF	AG	CC	PSNR	MSSSIM	DF
DDFM	**3.452** **±** **0.03**	4.376 ± 0.06	0.269 ± 0.001	4.940 ± 0.70	**0.904** **±** **0.002**	**64.103** **±** **0.66**	**0.408** **±** **0.002**	5.976 ± 1.06
EMMA	2.943 ± 0.05	5.364 ± 0.11	0.328 ± 0.001	7.373 ± 1.29	0.900 ± 0.002	62.419 ± 0.54	0.315 ± 0.002	8.611 ± 1.88
IFCNN	2.807 ± 0.04	4.600 ± 0.06	0.319 ± 0.001	8.280 ± 1.91	0.899 ± 0.002	63.435 ± 0.39	0.336 ± 0.002	10.089 ± 2.92
NSST-PAPCNN	2.818 ± 0.05	5.089 ± 0.08	**0.338** **±** **0.001**	7.683 ± 1.65	0.895 ± 0.003	62.934 ± 0.58	0.313 ± 0.002	9.059 ± 2.38
RCGAN	2.497 ± 0.05	5.184 ± 0.06	0.232 ± 0.001	5.134 ± 0.70	0.900 ± 0.002	63.642 ± 0.64	0.400 ± 0.002	6.092 ± 1.01
U2Fusion	2.530 ± 0.04	4.847 ± 0.06	0.224 ± 0.001	5.878 ± 0.62	0.898 ± .003	63.604 ± 0.54	0.392 ± 0.002	6.945 ± 0.88
GIFNet	2.603 ± 0.05	5.023 ± 0.06	0.228 ± 0.001	**10.366** **±** **1.87**	0.894 ± 0.002	62.513 ± 0.54	0.329 ± 0.002	**12.282** **±** **2.79**
FreeMMIF								
α_*b*_ = 1.0, α_*s*_ = 1.0	2.649 ± 0.10	**5.768** **±** **0.03**	0.245 ± 0.003	**9.888** **±** **1.96**	0.895 ± 0.002	62.151 ± 0.37	0.327 ± 0.002	**11.764** **±** **2.57**
α_*b*_ = 1.5, α_*s*_ = 0.5	**3.166** **±** **0.07**	5.325 ± 0.05	**0.364** **±** **0.001**	6.027 ± 1.46	0.896 ± 0.003	**64.539** **±** **0.19**	**0.401** **±** **0.005**	7.089 ± 1.93
α_*b*_ = 1.4, α_*s*_ = 0.6	3.087 ± 0.02	5.650 ± 0.04	0.296 ± 0.001	6.079 ± 1.22	0.898 ± 0.003	64.181 ± 0.22	**0.401** **±** **0.003**	7.288 ± 1.64
α_*b*_ = 1.3, α_*s*_ = 0.7	2.975 ± 0.03	**5.696** **±** **0.05**	0.259 ± 0.001	5.972 ± 0.91	0.901 ± 0.002	63.674 ± 0.38	0.394 ± 0.002	7.286 ± 1.27
α_*b*_ = 1.2, α_*s*_ = 0.8	2.796 ± 0.04	5.615 ± 0.05	0.252 ± 0.001	6.066 ± 0.67	0.903 ± 0.002	63.597 ± 0.49	0.380 ± 0.002	7.458 ± 1.00
α_*b*_ = 1.1, α_*s*_ = 0.9	2.888 ± 0.06	5.676 ± 0.04	0.278 ± 0.001	6.490 ± 0.81	**0.909** **±** **0.002**	63.986 ± 0.65	0.364 ± .002	7.972 ± 1.12

Quantitative comparison of CT-MRI fusion performance on the Harvard Medical Dataset. The values in bold indicate the best results for each evaluation metric; where two values are highlighted, they denote the best and the second-best results, respectively. For all metrics, a higher value indicates better performance.

As shown in Table 1, FreeMMIF with different parameters achieves the best values across the majority of metrics, including entropy (EN), mutual information (MI), average gradient (AG), and visual information fidelity (VIF). Specifically, the optimal scores in EN and MI demonstrate that, compared to other methods, FreeMMIF effectively extracts more original image information from both CT and MRI modalities. The highest AG and definition (DF) values confirm that our adaptive feature re-weighting strategy successfully preserves texture details and edge information, avoiding the smoothing effects often seen in GAN-based methods.

To rigorously validate our quantitative findings, we present the mean and standard deviation for all IQA metrics in Tables 1, 2. Furthermore, we conducted paired sample t-tests to evaluate the statistical significance of the performance differences between FreeMMIF and competitive baseline methods. The paired t-tests generally yield *p*-values strictly less than 0.05, demonstrating that the improvements achieved by our method are statistically significant at the 95% confidence level.

**Table 2:** Quantitative comparison of PET-MRI fusion performance.

	MI	EN	VIF	AG	CC	PSNR	MSSSIM	DF
DDFM	**3.755** **±** **0.07**	5.325 ± 0.41	0.389 ± .001	7.141 ± 0.81	0.903 ± .001	63.638 ± 0.59	0.261 ± .006	8.835 ± 1.58
EMMA	2.551 ± 0.05	5.112 ± 0.30	0.366 ± .001	9.729 ± 1.88	0.892 ± .001	63.565 ± 0.50	0.229 ± .006	11.872 ± 2.91
IFCNN	2.752 ± 0.03	5.410 ± 0.41	0.369 ± .001	**11.372** **±** **2.42**	0.889 ± .001	64.094 ± 0.37	0.223 ± .006	**14.019** **±** **4.56**
NSST-PAPCNN	3.390 ± 0.08	5.599 ± 0.44	**0.425** **±** **.001**	10.938 ± 2.16	0.892 ± .001	**64.389** **±** **0.80**	0.211 ± .006	**13.701** **±** **4.17**
RCGAN	2.571 ± 0.03	6.002 ± 0.32	0.315 ± .000	6.879 ± 0.74	0.898 ± .001	64.173 ± 0.41	0.254 ± .005	7.973 ± 1.18
U2Fusion	2.624 ± 0.02	5.507 ± 0.32	0.280 ± .000	6.854 ± 0.74	0.887 ± .001	63.380 ± 0.50	0.288 ± .006	8.237 ± 1.26
GIFNet	2.516 ± 0.02	5.615 ± 0.34	0.262 ± .000	10.419 ± 1.49	0.874 ± .001	62.421 ± 0.35	0.240 ± .006	12.099 ± 2.28
FreeMMIF								
α_*b*_ = 1.0, α_*s*_ = 1.0	**3.479** **±** **0.20**	6.379 ± 0.14	0.392 ± .001	**10.981** **±** **2.14**	0.890 ± .001	63.199 ± 0.26	0.223 ± .006	13.479 ± 3.99
Secondbest α_*b*_ = 1.5, α_*s*_ = 0.5	3.291 ± 0.12	6.208 ± 0.12	0.391 ± .000	4.648 ± 0.29	**0.919** **±** **.001**	62.697 ± 0.43	**0.348** **±** **.005**	5.760 ± 0.43
α_*b*_ = 1.4, α_*s*_ = 0.6	3.289 ± 0.11	6.366 ± 0.12	0.375 ± .000	5.795 ± 0.42	0.903 ± .001	62.780 ± 0.40	**0.290** **±** **.005**	7.181 ± 0.69
α_*b*_ = 1.3, α_*s*_ = 0.7	3.231 ± 0.11	**6.380** **±** **0.12**	0.363 ± .001	7.080 ± 0.68	**0.904** **±** **.001**	63.124 ± 0.35	0.264 ± .005	8.716 ± 1.20
α_*b*_ = 1.2, α_*s*_ = 0.8	3.219 ± 0.13	6.337 ± 0.12	0.368 ± .001	8.457 ± 1.04	0.899 ± .001	63.839 ± 0.30	0.248 ± .005	10.354 ± 1.89
Best α_*b*_ = 1.1, α_*s*_ = 0.9	3.453 ± 0.16	**6.434** **±** **0.10**	**0.394** **±** **.001**	10.021 ± 1.46	0.893 ± .001	**64.524** **±** **0.65**	0.233 ± .005	12.258 ± 2.59

Quantitative comparison of PET-MRI fusion performance. The values in bold indicate the best results for each evaluation metric; where two values are highlighted, they denote the best and the second-best results, respectively. For all metrics, a higher value indicates better performance.

### 4.3 Results of PET-MRI medical image fusion

This section evaluates the performance of FreeMMIF in fusing PET and MRI images, focusing on the preservation of functional metabolic information and anatomical structural details.

#### 4.3.1 Qualitative comparisons

**Figure 6:**
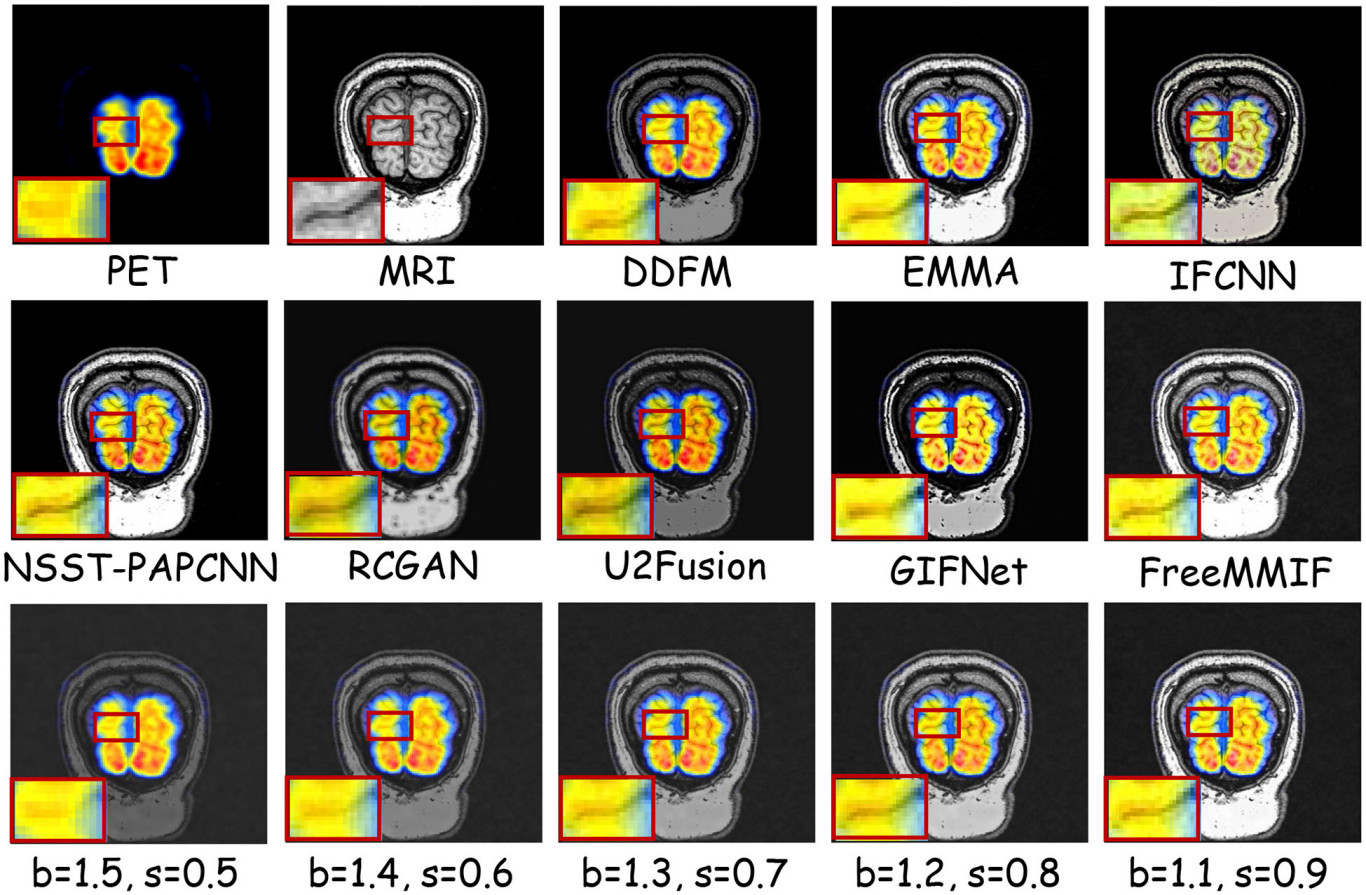
Visual comparison of PET-MRI fusion results.

**Figure 7:**
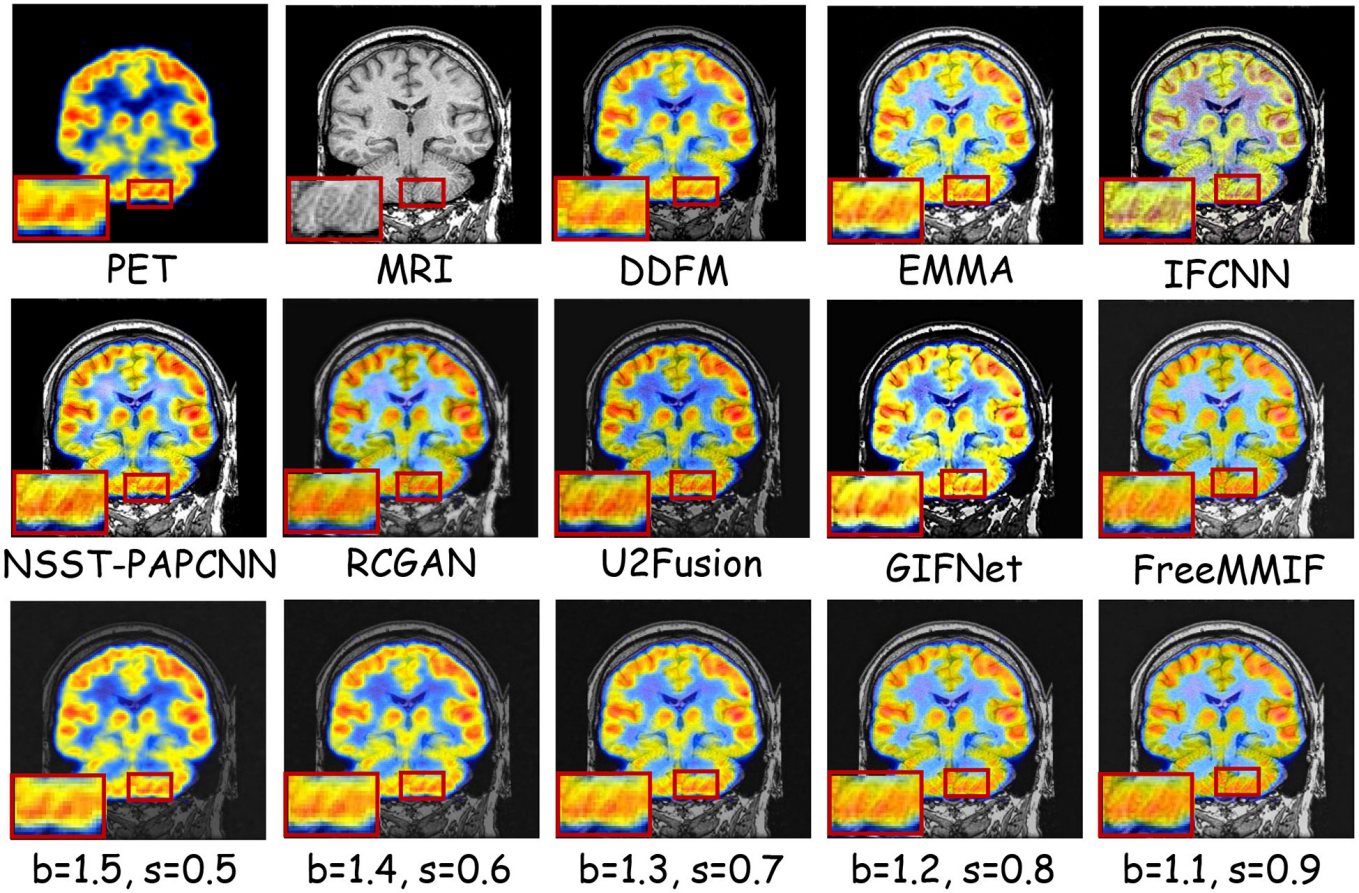
Visual comparison of PET-MRI fusion results.

We compare the fusion results of FreeMMIF with seven state-of-the-art methods on the PET-MRI fusion task. Figures 6, 7 visually present the fused images obtained by different methods.

As shown in Figure 6, existing methods, such as IFCNN and U2Fusion, often struggle to maintain the spectral fidelity of PET images, resulting in washed-out colors that obscure regions of high metabolic activity. Conversely, methods like LRFNet tend to suppress the background texture of MRI images, leading to a loss of spatial context information. In contrast, FreeMMIF effectively preserves the vibrant color information representing metabolic activity while seamlessly integrating high-frequency texture details from the MRI modality. The fused images exhibit clear anatomical boundaries and accurate functional localization, which is crucial for lesion identification.

Furthermore, Figure 7 demonstrates the versatility and robustness of FreeMMIF across different scanning scenarios. Unlike traditional methods that rely on fixed fusion rules, FreeMMIF allows for the dynamic adjustment of information weights. By tuning the instruction-aware parameters (e.g., from α_*b*_ = 1.5, α_*s*_ = 0.5 to α_*b*_ = 1.1, α_*s*_ = 0.9), the model generates a spectrum of distinct fusion results. This capability enables physicians to highlight either metabolic intensity or anatomical structure according to specific clinical requirements, thereby avoiding the “one-size-fits-all” limitation and ensuring accurate diagnostic results in various clinical settings.

#### 4.3.2 Quantitative comparisons

We quantitatively evaluate the performance of FreeMMIF using eight standard metrics. Table 2 presents the quantitative comparison results between FreeMMIF and the other seven fusion methods on the PET-MRI dataset.

As shown in Table 2, FreeMMIF consistently outperforms other fusion methods. Specifically, FreeMMIF with different settings achieves the highest values in MI and EN metrics . The high scores in MI and EN indicate that our method effectively transfers a significant amount of complementary information from both source modalities into the fused image. Additionally, the higher correlation coefficient (CC) and peak signal-to-noise ratio (PSNR) values confirm that the fused images possess excellent visual quality and edge sharpness, validating the effectiveness of the proposed adaptive feature re-weighting mechanism in preserving diagnostically relevant details without introducing artifacts.

### 4.4 Results of interactive medical image fusion

**Figure 8:**
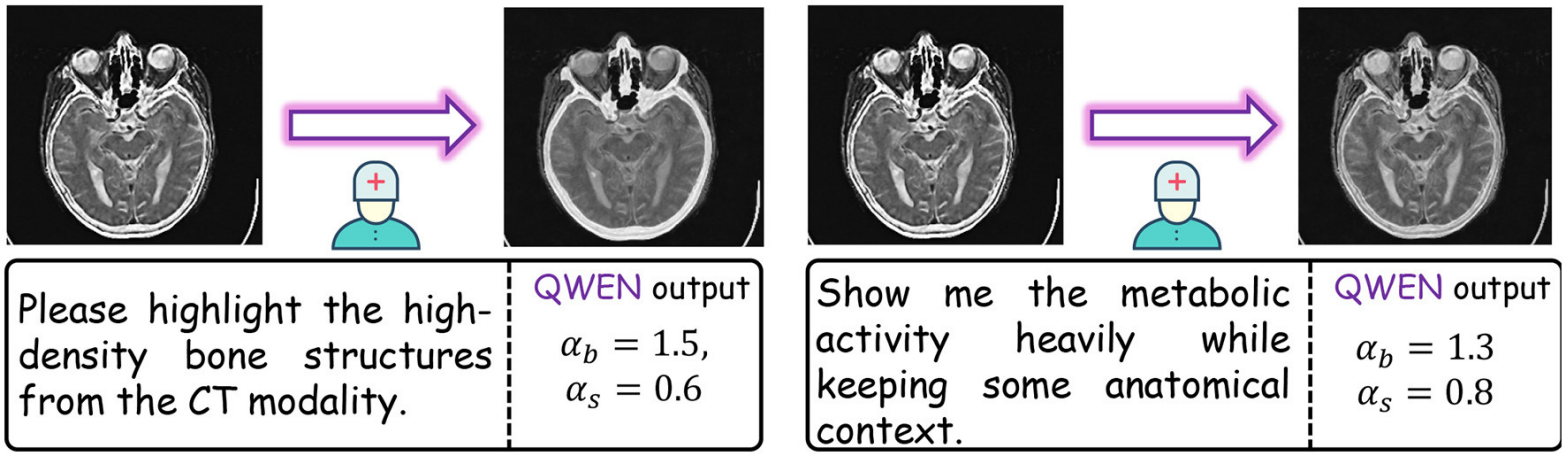
Illustration of the interactive fusion process.

In this section, we validate the effectiveness of the proposed interactive mechanism. Figure 8 illustrates the interactive fusion process where natural language instructions are translated into control parameters by the VLM to modulate the fusion results.

To facilitate this, we utilize a specialized prompt template labeled “Instruction for Clinical Intent Parameter Translation,” which explicitly defines the mapping logic between clinical keywords and the numerical modulation coefficients (α_*b*_, α_*s*_) to guide the VLM's reasoning. As shown in Figure 8, the framework effectively bridges the gap between abstract clinical intents and concrete model parameters. When a physician inputs a structure-oriented instruction such as “Please highlight the high-density bone structures from the CT modality,” the VLM interprets this intent and outputs a higher backbone scaling factor (α_*b*_ = 1.5) and a lower skip connection factor (α_*s*_ = 0.6). Consequently, the generated fusion image exhibits enhanced bone structures and clear anatomical boundaries, which are essential for structural diagnosis.

Conversely, when the instruction shifts to a functional focus, such as “Show me the metabolic activity heavily while keeping some anatomical context,” the VLM adjusts the weights to α_*b*_ = 1.3 and α_*s*_ = 0.8. The resulting image prioritizes the intensity of the metabolic regions while maintaining sufficient anatomical context for localization. This experiment confirms that FreeMMIF accurately comprehends varying clinical requirements and dynamically adapts the fusion strategy to produce preference-aligned results, significantly enhancing the flexibility and interpretability of the computer-aided diagnosis process.

Instruction for Clinical Intent Parameter Translation

- **Task Definition:** You are a parameter translator for the FreeMMIF medical fusion model. Analyze the user's clinical intent and output two control parameters.
- **Parameter Logic:**
  **– α_*b*_** **(Backbone scale):** High (1.3–1.5) favors *CT/Bone/Structure*. Low (1.0–1.2) favors *MRI/Soft Tissue*.
  **– α_*s*_** **(Skip scale):** Low (0.5–0.7) when α_*b*_ is high. High (0.8–1.0) favors *detailed texture/functional info*.
- **Formatting Rules:** Your output **MUST** be a JSON object containing only the values: {“alpha_b”: <value>, “alpha_s”: <value>}. No extra words or explanation.

**Figure 9:**
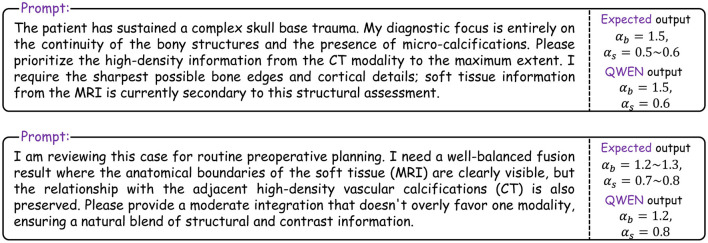
Evaluation of the language-mediated inference module using realistic, free-form clinical instructions.

To verify the system's robustness in real-world scenarios, we further evaluated the language-mediated inference module using realistic, free-form clinical instructions. Unlike simplified demonstration templates, clinical language is often nuanced and complex. As illustrated in Figure 9, our tests demonstrated that the VLM-based parameter translator consistently and accurately mapped complex, unconstrained requests to the appropriate expected ranges for the control coefficients (α_*b*_, α_*s*_). This confirms that FreeMMIF does not rely on rigid keyword matching, but genuinely understands clinical intent to drive the fusion process, thereby improving workflow efficiency.

While a simpler rule-based mechanism (e.g., keyword matching) might suffice for basic commands, we deliberately employ a VLM to bridge the semantic gap inherent in real-world clinical discourse. Clinical language often involves complex syntax, implicit clinical goals, and negations that deceive shallow parsing methods. In contrast, the VLM effectively comprehends the overarching semantic negation and context, ensuring the generated parameters (α_*b*_, α_*s*_) accurately reflect the true diagnostic intent. This deep semantic reasoning makes the VLM indispensable for a truly flexible and reliable interactive clinical tool.

### 4.5 Ablation studies

This section conducts extensive ablation studies to validate the effectiveness of the proposed frequency-aware adaptive feature re-weighting (FA-AFRM) module and the VLM-guided pseudo-ground truth construction strategy.

#### 4.5.1 Effectiveness of FA-AFRM

**Figure 10:**
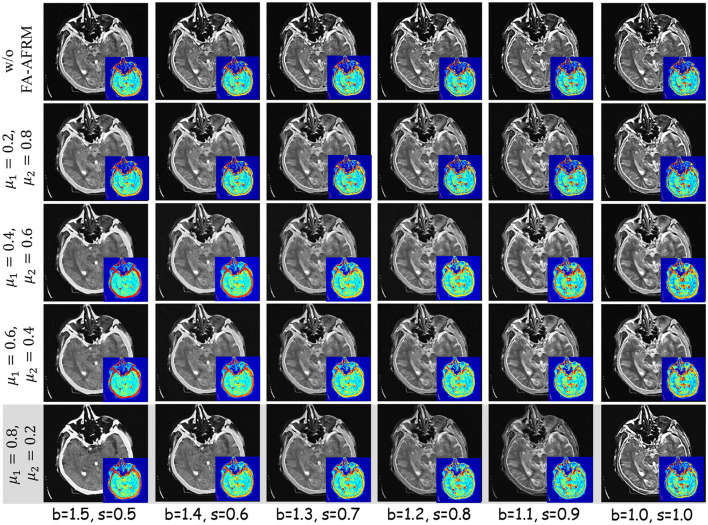
Visual ablation study showing the impact of the FA-AFRM module and different pseudo-ground truth mixing ratios.

We first verify the contribution of the FA-AFRM module by comparing the complete FreeMMIF model with a baseline variant (w/o FA-AFRM) where the feature modulation is removed, and the network operates as a standard diffusion model. Table 3 presents the quantitative results on the CT-MRI dataset. As shown in Table 3, the removal of FA-AFRM leads to a decline in key metrics; specifically, AG drops from 9.888 to 8.982, and EN decreases from 5.768 to 5.456. These results indicate that the FA-AFRM module effectively enhances the model's ability to preserve high-frequency details and information content by dynamically adjusting the feature weights in the frequency and spatial domains. Visual comparisons in Figure 10 further confirm that the model with FA-AFRM generates fused images with sharper edges and richer texture details compared to the baseline.

**Table 3:** Ablation study on the effectiveness of the FA-AFRM module.

	MI	EN	VIF	AG	CC	PSNR	MSSSIM	DF
w/o FA-AFRM	2.643	5.456	**0.249**	8.982	**0.899**	**62.784**	**0.331**	10.590
w FA-AFRM	**2.649**	**5.768**	0.245	**9.888**	0.895	62.151	0.327	**11.764**

The values in bold indicate the best results for each evaluation metric; where two values are highlighted, they denote the best and the second-best results, respectively. For all metrics, a higher value indicates better performance.

#### 4.5.2 Impact of pseudo-ground truth mixing ratios

We further investigate the impact of the mixing ratios μ_1_ and μ_2_ used during the pseudo-ground truth construction phase. This mixing strategy is crucial for mitigating potential biases or instabilities inherent in the VLM's selection process. Table 4 and Figure 10 illustrate the performance variations under different mixing configurations.

**Table 4:** Quantitative ablation analysis of the pseudo-ground truth mixing ratios.

	MI	EN	VIF	AG	CC	PSNR	MSSSIM	DF
w/o FA-AFRM	2.706	5.796	0.254	8.982	0.899	63.236	0.354	10.586
μ_1_ = 0.2, μ_2_ = 0.8	2.964	5.644	0.284	**9.026**	0.904	63.134	0.354	**10.648**
μ_1_ = 0.4, μ_2_ = 0.6	**3.020**	**5.810**	**0.298**	8.799	**0.905**	**63.876**	**0.389**	10.434
μ_1_ = 0.6, μ_2_ = 0.4	3.011	**6.000**	0.297	8.648	0.904	63.791	0.387	10.311
μ_1_ = 0.8, μ_2_ = 0.2	**3.166**	5.768	**0.364**	**9.888**	**0.909**	**64.539**	**0.401**	**11.764**

The values in bold indicate the best results for each evaluation metric; where two values are highlighted, they denote the best and the second-best results, respectively. For all metrics, a higher value indicates better performance.

As shown in Table 4, increasing the weight of the source images (μ_1_) generally improves the fusion quality. The configuration with μ_1_ = 0.8 and μ_2_ = 0.2 achieves the best performance across multiple metrics, yielding the highest MI of 3.166 and PSNR of 64.539. This suggests that prioritizing the original source information during training effectively neutralizes any potential semantic bias introduced by the VLM, providing more robust supervision than relying heavily on the algorithm-generated candidates. Figure 10 visually corroborates this finding, as μ_1_ increases from 0.2 to 0.8, the fused results exhibit clearer structural definitions and reduced artifacts. Consequently, we adopt μ_1_ = 0.8 and μ_2_ = 0.2 as the optimal setting for our final model to ensure high-fidelity fusion results. The ablation results confirm that using source images as the primary structural anchors provides a robust foundation for training. This anchor-based approach, combined with weight-driven VLM alignment, ensures that the model can dynamically adjust fusion results while maintaining high fidelity to the source modalities, effectively overcoming the limitations of missing ground truth in this field.

## 5 Conclusions

This paper introduces FreeMMIF, an interactive multimodal medical image fusion framework that leverages an instruction-aware diffusion model to achieve language-driven controllable fusion. FreeMMIF generates fusion results with varying information dominance from a single pair of source images and supports text-controlled dynamic adjustment based on user prompts. Its training procedure employs a novel VLM-guided pseudo-labeling strategy, which selects optimal candidates from representative methods to obtain preliminary prior knowledge. Existing methods typically yield a static solution oriented towards a single optimization objective, failing to accommodate diverse clinical diagnostic requirements. To address this, we propose an adaptive feature re-weighting mechanism tailored for medical image fusion. This mechanism enables the diffusion model to dynamically balance high-frequency and low-frequency information from different modalities during the generation process. Extensive experiments demonstrate that FreeMMIF achieves state-of-the-art performance in both objective metrics and visual quality. More importantly, the framework achieves precise semantic alignment with physician instructions, providing a highly interpretable and interactive tool that shows strong potential to enhance the reliability and flexibility of computer-aided diagnosis workflows in future clinical evaluations.

Despite these advantages, our framework has certain limitations that warrant future research. The primary limitation lies in the VLM-guided pseudo-ground truth construction during the training phase. Because the VLM acts as a selector from a candidate pool generated by existing static methods, its selection quality is upper-bounded by this pool. In rare failure cases–such as highly atypical pathologies where all baseline methods fail to produce adequate fusions–the VLM may be forced to select a suboptimal target. Although our dynamic mixing strategy mitigates this by strongly anchoring the target to the original source images, future work could explore end-to-end, zero-shot generative constraints that completely bypass the reliance on baseline candidate pools.

## Data Availability

The original contributions presented in the study are included in the article/supplementary material, further inquiries can be directed to the corresponding author.
